# Different Heschl’s Gyrus Duplication Patterns in Deficit and Non-deficit Subtypes of Schizophrenia

**DOI:** 10.3389/fpsyt.2022.867461

**Published:** 2022-06-16

**Authors:** Tsutomu Takahashi, Daiki Sasabayashi, Yoichiro Takayanagi, Atsushi Furuichi, Haruko Kobayashi, Kyo Noguchi, Michio Suzuki

**Affiliations:** ^1^Department of Neuropsychiatry, Graduate School of Medicine and Pharmaceutical Sciences, University of Toyama, Toyama, Japan; ^2^Research Center for Idling Brain Science, University of Toyama, Toyama, Japan; ^3^Arisawabashi Hospital, Toyama, Japan; ^4^Department of Radiology, Graduate School of Medicine and Pharmaceutical Sciences, University of Toyama, Toyama, Japan

**Keywords:** Heschl’s gyrus, schizophrenia, negative symptoms, deficit subtype, early neurodevelopment

## Abstract

Deficit syndrome schizophrenia is a characteristic subtype defined by persistent negative symptoms and poor functional outcomes; however, the biological mechanisms underlying this specific subtype have not yet been elucidated in detail. The present magnetic resonance imaging study examined the prevalence of duplicated Heschl’s gyrus (HG), a potential neurodevelopmental marker, in schizophrenia patients with (*N* = 38) and without (*N* = 37) the deficit syndrome. The prevalence of the HG duplication pattern bilaterally was higher in the whole schizophrenia group than in 59 matched healthy controls. Furthermore, the prevalence of right HG duplication was significantly higher in the deficit schizophrenia group than in the non-deficit schizophrenia group. The HG pattern in schizophrenia was not associated with clinical variables, including illness duration, medication, and symptom severity, while right HG duplication correlated with higher scores for Proxy for the Deficit Syndrome. The present results suggest that the prominent neurodevelopmental pathology associated with gyral formation of HG may contribute to enduring negative symptomatology in schizophrenia.

## Introduction

Schizophrenia is characterized by substantial clinical and biological heterogeneity, where negative symptoms are a core component that mainly account for long-term disability and poor functional outcomes in patients with the disorder (1, 2). The deficit form of schizophrenia (D-Sz), a well-defined clinical subgroup independent of the DSM (3) or ICD (4) subtype classification, is characterized by primary (i.e., not secondary to other factors, such as positive symptoms, comorbid depression, and extrapyramidal side effects) and enduring negative symptoms (5–7). The DSM/ICD subtypes of schizophrenia based on symptom profiles (e.g., paranoid, disorganized, and undifferentiated) have been eliminated because the subtype classification changes with time and cannot estimate their outcomes (8). On the other hand, the D-Sz defined by established classification tools {i.e., the Schedule for the Deficit Syndrome (9) or the Proxy for the Deficit Syndrome [PDS; (10)]} has a subtype stability over time and rather homogeneous outcome (7). While the etiological factors associated with D-Sz have not yet been identified, neuroimaging evidence of enhanced interregional cortical coupling (11) and altered gross brain morphology (e.g., gyrification patterns) (12, 13) specifically in D-Sz appear to support its prominent neurodevelopmental pathology. Therefore, brain morphological characteristics associated with early neurodevelopmental abnormalities, which likely exist at illness onset (14), may be a prognostic biomarker of worse long-term functioning in schizophrenia.

Recent magnetic resonance imaging (MRI) studies on schizophrenia revealed an increased prevalence of a duplicated Heschl’s gyrus (HG) pattern (15–17), which may reflect the development of an anomalous cytoarchitecture *in utero* (18, 19). This alteration in the gross brain morphology has been detected irrespective of illness stages [i.e., high-risk status (17), both first-episode (15) and chronic (16) stages] and the medication status (15–17) of schizophrenia. Furthermore, these studies suggested that the duplicated HG pattern was associated with severe prodromal symptomatology (17), but rather mild positive psychotic symptoms after onset (15, 16), as well as prominent verbal fluency deficit (17). These findings likely support the possibility that the HG gyrification pattern may contribute to specific clinical syndrome in schizophrenia. In addition, it has been suggested that the duplicated pattern of the HG, which participates in emotional processing (20), is associated with regional brain hypoactivity (21). It may be thus hypothesized that the patients with D-Sz, who are characterized by persistent negative symptoms, have an increased prevalence of HG duplication, but no MRI studies to date have specifically examined the HG duplication patterns in D-Sz.

Therefore, we herein used MRI to investigate and compare duplicated HG patterns in the D-Sz and non-deficit subtype of schizophrenia (ND-Sz) with those in matched healthy controls. We had previously explored brain characteristics of our D-Sz cohort and found gross morphological changes associated with early neurodevelopment [e.g., small adhesio interthalamica, altered distribution of the orbitofrontal sulcogyral pattern; (12)] and gray matter reduction in the insular cortex (unpublished data). The present study aimed to further expand these studies to HG duplication pattern, a recently identified early neurodevelopmental marker. Based on previous findings showing HG duplication in schizophrenia as a stable neurodevelopmental marker (15–17) and a putative prominent neurodevelopmental pathology in its deficit subtype (11–13), we predicted that the prevalence of HG duplication may be higher in patients with schizophrenia, particularly those with D-Sz. Furthermore, we investigated the relationships between the HG pattern and the clinical characteristics of patients in the different subgroups.

## Materials and Methods

### Participants

Thirty-eight patients with D-Sz, 37 with ND-Sz, and 59 healthy control subjects were enrolled in the present study (Table 1). We have previously investigated other brain structures (i.e., midline brain structures, orbitofrontal surface morphology) in this cohort (12), but this is the first study that specifically examined the relationship between the HG patterns and D-Sz using our data. However, the majority of the healthy controls and 51/75 schizophrenia patients examined in the present study had been included in our previous studies on HG patterns in the schizophrenia spectrum (16) and the volume-by-gyrification relationship of the HG in first-episode schizophrenia (15).

**TABLE 1:** Sample characteristics of subjects in the present study.

	Controls (*N* = 59)	Whole Sz (*N* = 75)	D-Sz (*N* = 38)	ND-Sz (*N* = 37)	Group comparisons between D- and ND-Sz groups*^a^*
Age (years)	26.1 ± 5.1	27.1 ± 6.8	27.1 ± 6.2	27.1 ± 7.5	*F* < 0.01, *p* = 0.984
Male/female	28/31	34/41	22/16	12/25	χ^2^ = 4.90, *p* = 0.027
Hand dominance (right/left/mix)	59/0/0	68/1/6	35/1/2	33/0/4	Fisher’s exact test, *p* = 0.551
Height (cm)	166.1 ± 8.0	163.8 ± 8.0	165.6 ± 8.3	162.0 ± 7.3	*F* = 3.81, *p* = 0.055
Parental education (years)	13.0 ± 2.5	12.5 ± 1.9	12.5 ± 2.0	12.5 ± 1.9	*F* < 0.01, *p* = 0.976
Personal education (years)	16.7 ± 2.4	13.5 ± 2.1	13.6 ± 2.1	13.3 ± 2.1	*F* = 0.32, *p* = 0.575
Onset age (years)	–	22.8 ± 5.9	23.0 ± 5.3	22.7 ± 6.6	*F* = 0.02, *p* = 0.876
Illness duration (years)	–	4.1 ± 4.8	4.1 ± 4.9	4.2 ± 4.9	*F* < 0.01, *p* = 0.954
Medication					
Duration (year)	–	2.5 ± 3.8	2.0 ± 2.9	3.1 ± 4.5	*F* = 1.79, *p* = 0.185
HPD equivalent dose (mg/day)	–	9.3 ± 8.5	8.3 ± 7.8	10.5 ± 9.0	*F* = 1.26, *p* = 0.265
Type (atypical/typical/mix)	–	49/21/3	24/11/1	25/10/2	Fisher’s exact test, *p* = 0.923
Total BPRS score	–	42.9 ± 12.6	36.5 ± 9.5	49.5 ± 12.0	*F* = 27.45, *p* < 0.001; ND-Sz > D-Sz
PDS score	–	−5.9 ± 4.5 (range, −15 to 3)	−1.8 ± 1.4 (range, −3 to 3)	−10.1 ± 1.8 (range, −15 to −8)	*F* = 504.04, *p* < 0.001; D-Sz > ND-Sz
SANS					Group-by-subscore interaction; *F*(4,292) = 5.25, *p* < 0.001
Blunted affect		14.4 ± 9.6	16.0 ± 8.8	12.7 ± 10.3	*Post hoc* comparison, *p* = 0.004; D-Sz > ND-Sz
Alogia	–	7.5 ± 5.0	8.0 ± 5.6	6.9 ± 4.3	–
Avolition-apathy	–	10.7 ± 4.9	10.8 ± 5.0	10.7 ± 4.8	–
Anhedonia-asociality	–	11.7 ± 7.0	10.6 ± 5.8	12.8 ± 7.9	–
Attention deficit	–	8.9 ± 4.6	7.5 ± 4.7	10.4 ± 4.1	*Post hoc* comparison, *p* = 0.011; D-Sz < ND-Sz
SAPS					Group-by-subscore interaction; *F*(3,219) = 12.58, *p* < 0.001
Hallucinations	–	9.3 ± 8.8	5.3 ± 7.5	13.5 ± 8.2	*Post hoc* comparison, *p* < 0.001; ND-Sz > D-Sz
Delusions	–	13.4 ± 10.3	8.1 ± 8.0	19.0 ± 9.6	*Post hoc* comparison, *p* < 0.001; ND-Sz > D-Sz
Bizarre behavior	–	5.0 ± 4.2	4.5 ± 4.0	5.5 ± 4.4	–
Positive formal thought disorder	–	5.2 ± 7.5	3.7 ± 5.6	6.8 ± 8.9	–

*Values show means ± SDs unless otherwise stated.BPRS, Brief Psychiatric Rating Scale; D-Sz, deficit schizophrenia; HPD, haloperidol; ND-Sz, non-deficit schizophrenia; PDS, Proxy for the Deficit Syndrome; SANS, Scale for the Assessment of Negative Symptoms; SAPS, Scale for the Assessment of Positive Symptoms; Sz, schizophrenia.^a^Df = 1, 73 for ANOVAs, except for SANS/SAPS comparisons.*

Briefly, schizophrenia patients meeting the ICD-10 research criteria (4) assessed *via* a structured clinical interview [the Comprehensive Assessment of Symptoms and History (22)] and chart review were recruited at Toyama University Hospital. Just prior to MRI being performed, clinical symptoms were examined by experienced psychiatrists using the Brief Psychiatric Rating Scale [BPRS (23)] and the Scale for the Assessment of Negative/Positive Symptoms [SANS/SAPS (24)]. As previously described (12, 13), patients were divided into the D and ND subgroups based on their PDS scores [i.e., blunted affect − (anxiety + guilty feelings + depressed mood + hostility items)] according to BPRS (10). Although the PDS score accurately reflects primary and enduring negative symptomatology in patients with schizophrenia (10, 25), we herein classified patients with the top (≥−3) and bottom (≤−8) 25[percentage] of PDS scores in our full schizophrenia dataset into the D and ND subgroups, respectively, to increase the likelihood of a correct classification (11).

Healthy subjects who had been screened using a questionnaire for a personal or family history of psychiatric disease in first-degree relatives (26) were selected from our previous studies (15, 16) based on matching to the patient group for demographic characteristics (e.g., age, sex, height, and parental education; Table 1).

All participants were physically healthy and screened for gross brain abnormalities using MRI. Exclusion criteria for patients and controls were as follows: (1) a lifetime history of serious head injury, seizure, neurological disease, or substance abuse disorder; (2) a history of electroconvulsive therapy; and (3) other medical conditions that may affect mental condition and/or brain functioning (e.g., thyroid dysfunction, steroid use, hypertension, and diabetes). The study protocol was approved by the Committee on Medical Ethics of the University of Toyama (No. I2013006). Written informed consent was obtained from all participants in accordance with the Declaration of Helsinki.

### Magnetic Resonance Imaging Acquisition and Processing

All participants underwent the same imaging protocol at Toyama University Hospital using a 1.5T Magnetom Vision scanner (Siemens, Erlangen, Germany) to obtain 160–180 contiguous T1-weighted 1-mm-thick sagittal slices *via* the 3D gradient-echo sequence FLASH. The following imaging parameters were used: TR/TE = 24 ms per 5 ms; flip angle = 40°; FOV = 256 mm; and matrix = 256 × 256 pixels, with a voxel size of 1.0 mm × 1.0 mm × 1.0 mm.

Brain images were reconstructed into 1-mm-thick contiguous coronal images that perpendicularly aligned with the anterior commissure-posterior commissure line using Dr. View software (Infocom, Tokyo, Japan).

### Evaluation of Heschl’s Gyrus Gyrification Patterns

**FIGURE 1:**
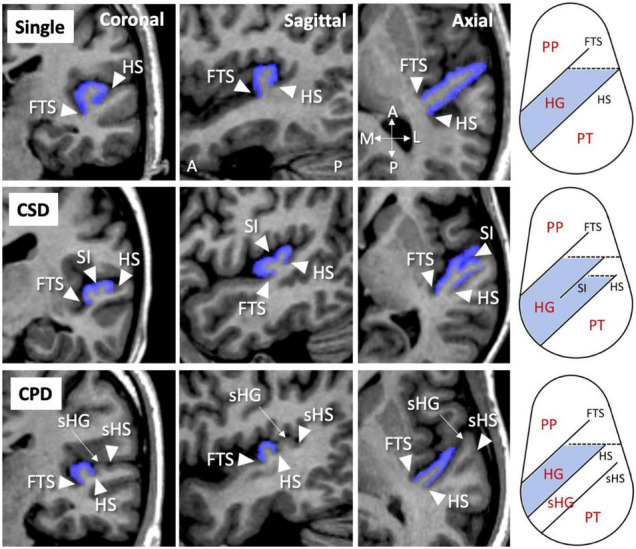
Different Heschl’s gyrus gyrification patterns (colored in blue) in the left hemisphere on MR images in three directions and a schematic diagram on axial directions. A, anterior; CPD, complete posterior duplication; CSD, common stem duplication; FTS, first transverse sulcus; HG, Heschl’s gyrus; HS, Heschl’s sulcus; L, lateral; P, posterior; M, medial; PP, planum polare; PT, planum temporale; sHG, second Heschl’s gyrus; sHS, second Heschl’s sulcus; SI, sulcus intermedius. Note that these patterns were presented also in our previous publications (15–17).

One rater (TT) assessed all MR images under blind conditions for subject identities. As previously described (15–17), the HG was classified into the single or duplicated pattern by referring to images in three directions; the duplicated pattern was further divided into partial duplication by the sulcus intermedius [common stem duplication (CSD)] or complete duplication accompanied by a second HG [complete posterior duplication (CPD)] (Figure 1). A second rater with no knowledge of the subjects’ identity (DS) independently assessed the HG patterns in a subset of randomly selected 15 brains. Intra- (TT) and inter-rater (TT and DS) reliabilities for the classification of HG were both higher than 0.83 (tested by Cronbach’s α).

### Statistical Analysis

A one-way analysis of variance (ANOVA) or the χ^2^ test (or Fisher’s exact test when more than 20[percentage] of cells had expected counts <5) was employed for group comparisons of demographic and clinical data.

We used the χ^2^ test to evaluate group differences (controls vs. whole schizophrenia, D- vs. ND-Sz) in HG gyrification pattern distributions in each hemisphere. Because the HG duplication was more prevalent in the patients especially for D-Sz, the odds ratio was calculated to estimate the relationship between HG duplication and relative risk of schizophrenia and its subtype. The relationships between HG patterns and clinical variables [onset age, illness duration, medication (dose, duration), total SANS/SAPS and BPRS scores, and PDS scores] were assessed by an analysis of covariance (ANCOVA) with age as a covariate, followed by Duncan’s test. *p* < 0.05 was considered to indicate a significant difference.

## Results

### Clinical Differences Between Schizophrenia Subgroups

The score for the blunted affect subscale was significantly higher in the D-Sz group than in the ND-Sz group, while delusions, hallucinations, and attention deficits were more severe in the ND group (Table 1). On the other hand, no significant differences were observed in the age of onset, duration of illness, or medication status between the subgroups. In accordance with the literature (27), the proportion of males was higher in the D-Sz group than in the ND-Sz group.

### Heschl’s Gyrus Pattern Distributions

**FIGURE 2:**
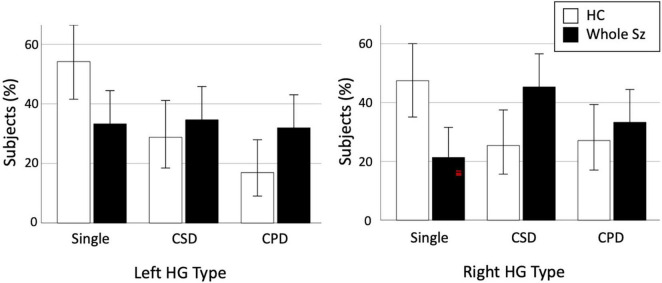
Prevalence of each Heschl’s gyrus (HG) gyrification pattern in healthy controls (HC) and whole schizophrenia (Sz). Error bars indicate 95[percentage] confidence intervals. CPD, complete posterior duplication; CSD, common stem duplication.

Comparisons between all patients with schizophrenia (*N* = 75) and healthy controls (*N* = 59) revealed significant differences for both the left (χ^2^ = 6.69, *p* = 0.035) and right (χ^2^ = 10.86, *p* = 0.004) hemispheres (Table 2). The prevalence of bilateral HG duplication patterns (CSD or CPD) was higher in patients than in the controls [left, χ^2^ = 5.90, *p* = 0.015, odds ratio = 2.37 (95[percentage] CI, 1.18–4.77); right, χ^2^ = 10.22, *p* = 0.001, odds ratio = 3.33 (95[percentage] CI, 1.59–7.03)] (Figure 2). We then examined right-handed subjects only because handedness distribution differed between the control and whole patient groups (Fisher’s exact test, *p* = 0.034); the results obtained remained essentially the same (Supplementary Material).

**TABLE 2:** Gyrification pattern of Heschl’s gyrus (HG) in the study participants.

			Right HG pattern [*N* (%)]		
	
		Single	CSD	CPD	Total
**Healthy controls**					
Left HG pattern [*N* ([percentage])]	Single	17 (28.8)	7 (11.9)	8 (13.6)	32 (54.2)
	CSD	7 (11.9)	5 (8.5)	5 (8.5)	17 (28.8)
	CPD	4 (6.8)	3 (5.1)	3 (5.1)	10 (16.9)
	Total	28 (47.5)	15 (25.4)	16 (27.1)	59 (100.0)
**Deficit schizophrenia**					
Left HG pattern [*N* ([percentage])]	Single	2 (5.3)	7 (18.4)	5 (13.2)	14 (36.8)
	CSD	2 (5.3)	5 (13.2)	5 (13.2)	12 (31.6)
	CPD	0 (0.0)	5 (13.2)	7 (18.4)	12 (31.6)
	Total	4 (10.5)	17 (44.7)	17 (44.7)	38 (100.0)
**Non-deficit schizophrenia**					
Left HG pattern [*N* ([percentage])]	Single	7 (18.9)	2 (5.4)	2 (5.4)	11 (29.7)
	CSD	2 (5.4)	9 (24.3)	3 (8.1)	14 (37.8)
	CPD	3 (8.1)	6 (16.2)	3 (8.1)	12 (32.4)
	Total	12 (32.4)	17 (45.9)	8 (21.6)	37 (100.0)

*CSD, common stem duplication; CPD, complete posterior duplication.*

**FIGURE 3:**
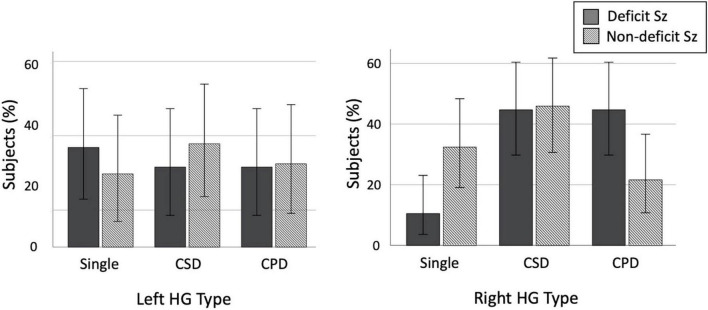
Prevalence of each Heschl’s gyrus (HG) gyrification pattern in patients with deficit and non-deficit schizophrenia (Sz). Error bars indicate 95[percentage] confidence intervals. CPD, complete posterior duplication; CSD, common stem duplication.

In subgroup comparisons between the D- and ND-Sz groups, a significant difference was only observed for the right hemisphere (left, χ^2^ = 0.50, *p* = 0.779; right, χ^2^ = 7.23, *p* = 0.027). The prevalence of right HG duplication was significantly higher in the D-Sz subgroup than in the ND-Sz subgroup [χ^2^ = 5.36, *p* = 0.021, odds ratio = 4.08 (95[percentage] CI, 1.22–13.42)] (Figure 3). When only patients with HG duplication patterns (i.e., CSD vs. CPD) were examined, a subgroup difference was not observed (left, χ^2^ = 0.07, *p* = 0.786; right, χ^2^ = 1.91, *p* = 0.167).

Furthermore, there was no significant effect of sex on HG duplication in the controls, the schizophrenia group as a whole, or each schizophrenia subgroup.

### Heschl’s Gyrus Pattern and Clinical Variables in Schizophrenia

The HG pattern did not affect the onset age, duration of illness, medications, or symptom ratings (total SANS, SAPS, and BPRS scores) in the D- and ND-Sz subgroups. As predicted by significant contribution of right HG duplication to the D-Sz (described above), in the schizophrenia group as a whole, patients with the right duplicated pattern had a significantly higher PDS score (i.e., higher characteristic tendency for the deficit subtype) than those with right single HG [*F*(1,72) = 4.66, *p* = 0.034; *post hoc* test, *p* = 0.034]. This difference in the PDS score was especially evident between patients with CPD (mean = −4.24, SD = 3.87) and those with the single pattern (mean = −8.00, SD = 3.90) [*F*(2,71) = 3.74, *p* = 0.029; *post hoc* test, *p* = 0.007]. On the other hand, the left HG pattern in the schizophrenia group as a whole did not affect these clinical variables.

## Discussion

While its preliminary nature with a small number of subjects, we demonstrated for the first time that prevalence of right HG duplication was higher in patients with D-Sz than in those with ND-Sz. This difference was not explained by demographic or clinical differences in these subgroups, except for the PDS score, which reflects the trait characteristics of deficit syndrome. These results suggest that prominent early neurodevelopmental abnormalities associated with sulcal formation during gestation may contribute to the characteristic clinical manifestations of D-Sz, such as enduring negative symptomatology and poor functional outcomes.

The present study demonstrated that the prevalence of the right HG duplication patterns was significantly higher in patients with schizophrenia, particularly those with the deficit subtype, than in healthy controls, while the HG duplication itself has been reported in approximately 30–50[percentage] of healthy adults (28–30). The functional significance of this anatomical variation currently remains unclear, but may reflect inter-individual differences in the cytoarchitectonic development of the primary auditory cortex *in utero* (18, 19); duplicated HG has been implicated in the development of learning disabilities (31, 32) and the inhibition of HG activity in auditory processes (21) after birth, even in non-clinical populations. HG is a primary brain region of auditory processing, but also participates in emotional processing (20) and social communication (33). Therefore, the core clinical features of D-Sz, such as persistent blunted affect and prominent cognitive deficits, particularly in social and verbal domains (7), may be partly attributed to neurodevelopmental abnormalities associated with HG sulcus formation. Indeed, our previous study on the early stages of psychosis (17) revealed a correlation between the HG duplication pattern and verbal fluency deficits, although it was evident between different duplication patterns (i.e., CSD vs. CPD) in the left hemisphere. Since brain gyrification may reflect regional neural connectivity (34), our hypothesis needs to be tested in a cohort with more detailed clinical assessments using the multimodal neuroimaging of brain connectivity/function. Potential clinical significance of different HG duplication patterns (CSD, CPD) should be also tested in such future studies.

The present results on HG duplication patterns were not influenced by illness chronicity or medication, supporting previous neuroimaging findings suggesting a prominent early neurodevelopmental pathology in D-Sz [e.g., small adhesio interthalamica (12) and gyrification pattern changes (12, 13)]. We failed to replicate our previous findings in larger and less confounded (i.e., first-episode) schizophrenia groups to show that the right CPD pattern specifically contributed to less severe positive symptomatology (15, 16); however, the right CPD pattern in the present cohort correlated with the trait characteristics of deficit syndrome (i.e., PDS score). In addition, the right lateralized group difference in the HG pattern in the present study may support right hemisphere dominance for emotion processing, particularly for negative emotional information (35), as well as D-Sz having severe and prolonged neurodevelopmental abnormalities. Right HG generally develops 1–2 weeks earlier than left HG during mid-to-late gestation (18) and is more complex (18, 29); therefore, gyral formation of HG in D-Sz may be more affected on the right hemisphere. Since a meta-analysis of gray and white matter volumes across various brain regions found no significant differences between D- and ND-Sz (36) and these volumetric data are affected by various confounding factors (e.g., illness chronicity and medication), a better predictive biomarker of the clinical subtype and course of schizophrenia may be gross brain morphology, which is strongly related to early neurodevelopment.

Several potential confounding factors in the present study need to be addressed. First, the present study was partly limited by its small sample size for each schizophrenia subgroup. Due to its reliability and stability, the PDS score has been widely used to classify patients into the D and ND subgroups in biological (11, 37–39) and clinical (40–42) studies (10, 25, 41, 43); however, a cross-sectional PDS rating does not have the capacity to directly assess enduring deficit-like features. Therefore, we excluded the ambiguous middle group for the PDS score (−8 to −3) from the current schizophrenia sample in an attempt to reduce potential false classifications, as in previous imaging studies (11–13), which further decreased the number of schizophrenic patients examined. Therefore, the present results need to be confirmed in a well-defined cohort of a large number of patients with D-Sz with clinical follow-up data and/or a semi-structured interview [e.g., the Schedule for the Deficit Syndrome (9)]. Second, the sex ratio significantly differed between the D- and ND-Sz subgroups in the present group, potentially reflecting the general characteristics of D-Sz [more males than females (27)]. However, this difference did not appear to significantly affect the results obtained because there was no significant sex effect on HG patterns. Third, we did not systematically assess the cognitive function of our cohort. Since HG duplication patterns have been implicated in prominent cognitive impairments in D-Sz, as described above, future studies on the gyrification–cognition relationship may provide a more detailed understanding of the pathophysiology of this specific schizophrenia subtype. Finally, although the present HG pattern classification by manual delineation on 1.5T MRI data was reliable (Cronbach’s α > 0.83), our results should be replicated using unbiased automatic analysis on higher resolution images.

In summary, this preliminary MRI study demonstrated that the prevalence of HG duplication was higher in patients with schizophrenia, particularly those with the deficit syndrome subtype who typically exhibit enduring negative symptomatology and poor functional outcomes. Although the HG duplication itself is observed in healthy subjects and there is considerable overlap on the HG pattern distribution between D- and ND-Sz subgroups, it is possible that, in combination with other brain characteristics, the HG pattern in the early stages of schizophrenia may have potential as a prognostic biomarker of worse long-term functioning.

## Data Availability Statement

The raw data supporting the conclusions of this article will be made available by the authors, without undue reservation.

## Ethics Statement

The studies involving human participants were reviewed and approved by the Committee on Medical Ethics of the University of Toyama. The patients/participants provided their written informed consent to participate in this study.

## Author Contributions

MS and TT conceived the idea and methodology of this study. TT conducted the statistical analyses and wrote the manuscript. DS and HK recruited the participants and were involved in clinical and diagnostic assessments. TT and DS analyzed the MRI data. KN provided the technical support for MRI scanning and data processing. AF managed the MRI and clinical data. MS and YT contributed to the writing and editing of the manuscript. All authors contributed to and approved the final manuscript.

## Conflict of Interest

The authors declare that the research was conducted in the absence of any commercial or financial relationships that could be construed as a potential conflict of interest.

## Publisher’s Note

All claims expressed in this article are solely those of the authors and do not necessarily represent those of their affiliated organizations, or those of the publisher, the editors and the reviewers. Any product that may be evaluated in this article, or claim that may be made by its manufacturer, is not guaranteed or endorsed by the publisher.
