# Supplementary material for: Different Heschl’s Gyrus Duplication Patterns in Deficit and Non-deficit Subtypes of Schizophrenia
Source: Front Psychiatry. 2022 Jun 16;13:867461. doi: 10.3389/fpsyt.2022.867461 (PMC9243379; doi:10.3389/fpsyt.2022.867461)
Supplement: Supplementary file 1 [file Table_1.DOCX]

Supplementary Material

**HG Pattern Distributions Only in Right-Handed Healthy Controls and Right-Handed Schizophrenia Patients.**

Comparisons between right-handed schizophrenia patients (*N* = 68) and healthy controls (*N* = 59) revealed significant differences for both the left (χ^2^ = 8.82, *p* = 0.012) and right (χ^2^ = 10.91, *p* = 0.004) hemispheres. The prevalence of bilateral Heschl’s gyrus duplication patterns (common stem duplication or complete posterior duplication) was higher in patients than in the controls [left, χ^2^ = 8.05, *p* = 0.005, odds ratio = 2.84 (95% CI, 1.37 to 5.91); right, χ^2^ = 10.30, *p* = 0.001, odds ratio = 3.48 (95% CI, 1.60 to 7.59)].
